# Electro-conversion of cumene into acetophenone using boron-doped diamond electrodes

**DOI:** 10.3762/bjoc.18.119

**Published:** 2022-09-07

**Authors:** Mana Kitano, Tsuyoshi Saitoh, Shigeru Nishiyama, Yasuaki Einaga, Takashi Yamamoto

**Affiliations:** 1 Department of Chemistry, Keio University, 3-14-1 Hiyoshi, Yokohama 223-8522, Japan; 2 International Institute for Integrative Sleep Medicine (WPI-IIIS), University of Tsukuba, 1-1-1 Tennodai, Tsukuba 305-8575, Japan

**Keywords:** aromatic alkyl, boron-doped diamond electrode, electrosynthesis, oxidation

## Abstract

A straightforward electro-conversion of cumene into acetophenone has been reported using boron-doped diamond (BDD) electrodes. This particular conversion is driven by the addition reaction of a cathodically generated hydroperoxide anion to an anodically generated cumyl cation, where the BDD’s wide potential window enables the direct anodic oxidation of cumene into the cumyl cation. Since electricity is directly employed as the oxidizing and reducing reagents, the present protocol is easy to use, suitable for scale-up, and inherently safe.

## Introduction

Selective oxidation of aromatic alkyl side chains is an important molecular transformation process to obtain various rubbers, resins, fine chemicals, and other industrial products [1–2]: terephthalic acid from *p*-xylene, cumene hydroperoxide/dicumyl peroxide/phenol from cumene, acetophenone from ethylbenzene, and others. Generally, molecular oxygen has been utilized in the straightforward oxidation of aromatic alkyls. However, since molecular oxygen is highly stable, activation of the molecular oxygen itself is necessary, which requires a specific catalyst and/or harsh conditions such as high temperature and pressure. Recent environmental and sustainable concerns lead to a growing demand for the development of greener oxidation processes. For example, even for the cumene process that involves the oxidation reaction of cumene, first reported in 1944 [3], a wide variety of catalytic systems are still being reported [4–12].

Electro-organic synthesis refers to an organic synthetic method combined with electrochemistry [13–14]. A striking feature in electro-organic synthesis is the use of electricity as a reagent, which allows to reduce the reagent waste to a minimum. Obviously, as this characteristic matches well with the increasing demands to realize a sustainable society. In electro-organic synthesis, electrode materials are one of the most significant parameters because reactions occur at the anode and/or cathode. Boron-doped diamond (BDD) is a relatively new electrode material [15–16] and shows a wide potential window, which can be applied to the transformation of compounds with high redox potentials. Therefore, BDD electrodes would enable a straightforward oxidation reaction of aromatic alkyls, which is difficult to achieve with other conventional electrode materials.

Herein, we report the straightforward electro-conversion of cumene, one of the most important and extensively investigated aromatic alkyls, by BDD electrodes. Acetophenone was obtained as the main product when BDD was used as the anode. The role of electrode materials was investigated with electrochemical measurements. Only the BDD anode with a wide potential window can oxidize cumene directly to afford a cumyl cation as the reaction intermediate. Furthermore, acetophenone is produced via cumene hydroperoxide, and this molecular conversion is found to proceed electrochemically.

## Results and Discussion

First, we carried out the electrolysis of cumene (**1**) in 0.1 M Bu_4_NBF_4_/MeCN under constant current conditions in an undivided beaker-type cell (Table 1, entry 1). The main product was acetophenone (**3**) and α-cumyl alcohol (**4**) was also obtained. When the anion of the supporting electrolyte was changed to the perchlorate ion (Table 1, entries 2–4), isolated yields of **3** were increased, in which using Et_4_NClO_4_ gave the best result. Next, the current density (*j*) and the amount of charge (*Q*; referring to mole of **1**) were investigated (Table 1, entries 5–7). As the isolated yield of **3** was not particularly improved by changing *j* and *Q*, we set the optimum conditions as *j* of 2.1 mA/cm^2^ and *Q* of 5 F. On the other hand, when the combination of anode and cathode was graphite/graphite or Ni/Ni (Table 1, entries 8 and 9), almost no acetophenone was obtained. Therefore, it is suggested that the BDD anode is essential in the electro-conversion of **1** into **3**, and the cathode material has no significant effect. Here, the low total yields would be due to the formation of highly polar compounds. We were not able to obtain them as isolated compounds. In addition, ^1^H NMR and FTIR spectra for the crude compound did not show characteristic peaks derived from carboxylic acid and amide.

**Table 1:** Electro-conversion of **1**.

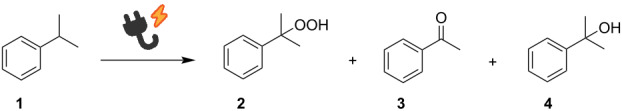									

Entry^a^	Anode	Cathode	Supporting electrolyte	*j* ^b^	*Q* ^c^	Isolated yields (%)			

						**1**	**2**	**3**	**4**

1	BDD	BDD	Bu_4_NBF_4_	2.1	5	n.d.	n.d.	18	14
2	BDD	BDD	Bu_4_NClO_4_	2.1	5	n.d.	1	27	9
3	BDD	BDD	LiClO_4_	2.1	5	n.d.	n.d.	19	13
4	BDD	BDD	Et_4_NClO_4_	2.1	5	trace	1	34	11
5	BDD	BDD	Et_4_NClO_4_	2.1	3	3	4	17	28
6	BDD	BDD	Et_4_NClO_4_	2.1	7.5	n.d.	n.d.	32	n.d.
7	BDD	BDD	Et_4_NClO_4_	1.05	5	5	2	23	26
8	graphite	graphite	Et_4_NClO_4_	2.1	5	4	n.d.	1	4
9	Ni	Ni	Et_4_NClO_4_	2.1	5	4	n.d.	n.d.	n.d.
10	BDD	graphite	Et_4_NClO_4_	2.1	5	trace	trace	33	5
11	graphite	BDD	Et_4_NClO_4_	2.1	5	5	n.d.	trace	20

^a^Reaction conditions: 1 mmol cumene (**1**), 5 mL MeCN, 0.1 M supporting electrolyte, undivided beaker-type cell, rt; ^b^current density (mA/cm^2^); ^c^amount of charge (F) referring to mole of **1**. n.d. = not detected.

**Figure 1:**
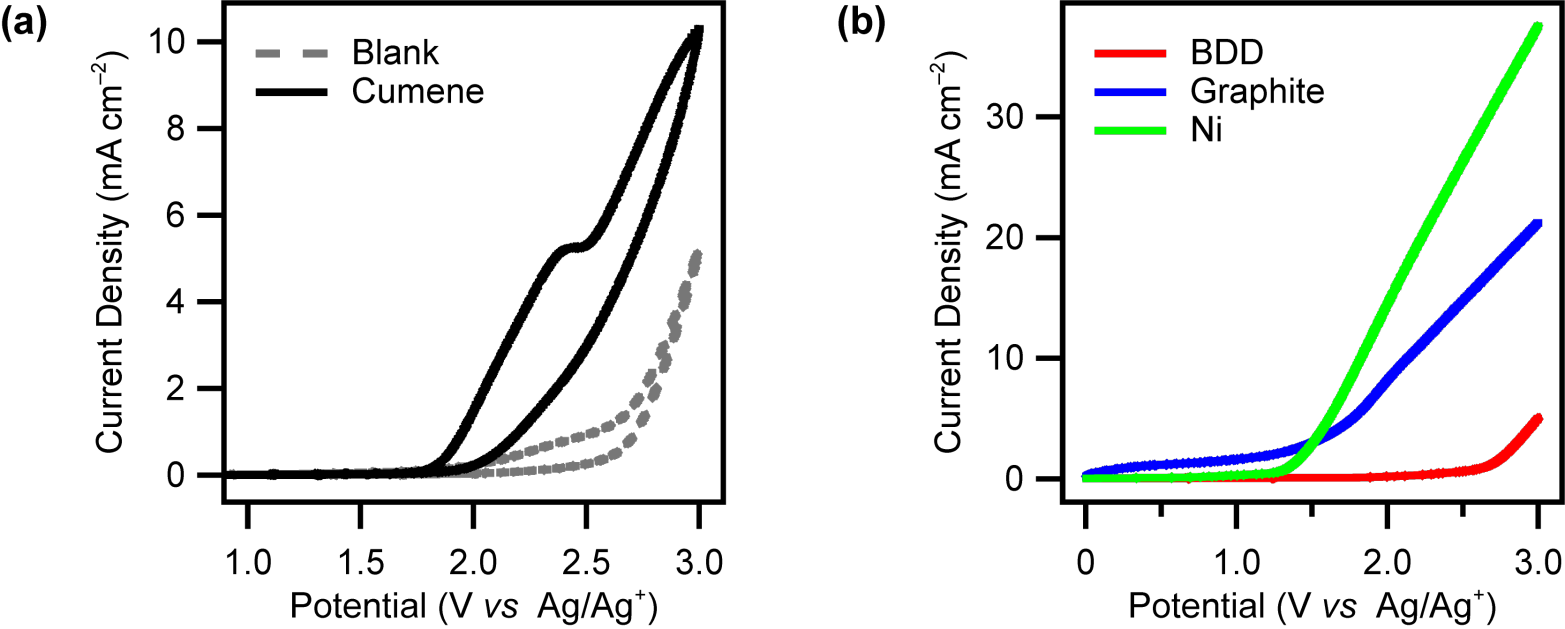
(a) Cyclic voltammograms of a BDD electrode in MeCN solution containing cumene (**1**; 5 mM) and Et_4_NClO_4_ (0.1 M). The gray dashed line shows the voltammogram in the solution without cumene. (b) Linear sweep voltammograms of BDD (red), graphite (blue), and Ni (green) electrodes in MeCN solution containing Et_4_NClO_4_ (0.1 M). Scan rate: 100 mV/s.

In order to clarify the role of the anode material, we carried out electrochemical measurements (Figure 1). Cyclic voltammetry was performed using BDD as a working electrode. A clear oxidation peak of **1** was observed at around 2.40 V (vs Ag/Ag^+^) (Figure 1a), which is comparable to a previous report using a Pt disk electrode [17]. On the other hand, no clear oxidation peak was observed when using graphite or Ni as a working electrode. This is because potential windows of graphite and Ni are too narrow to oxidize **1** directly, as can be seen in Figure 1b. Overall, the electrochemical measurements indicate the BDD’s wide potential window enables direct oxidation of **1** to produce a key reaction intermediate to afford **3**.

A series of electrolysis experiments was performed to propose a reaction mechanism (Table 2). First, we carried out the electrolysis of **1** in MeCN–MeOH to confirm whether the reaction intermediate is a radical or cationic species (Table 2, entry 1). As a result, methyl cumyl ether, a methoxy adduct to the benzyl position of **1**, was obtained as the main product in 21% yield. Therefore, it is indicated that the reaction intermediate is the cumyl cation. Second, we carried out the electrolysis of **1** in MeCN–H_2_O to confirm whether the oxygen source is dissolved oxygen or residual water. When dehydrated MeCN was used, **3** was obtained as the main product (Table 2, entry 2). On the other hand, the isolated yield of **3** was decreased by the addition of H_2_O (Table 2, entries 3 and 4). This is probably because the addition of H_2_O promoted the generation of hydroxyl radicals, and a decomposition reaction became dominant. These results indicated that the oxygen source is not residual water in MeCN, but dissolved oxygen. The role of dissolved oxygen was further investigated. As the reaction did not proceed without electricity, it is suggested that the superoxide generated on the cathode is involved in the reaction, rather than dissolved molecular oxygen itself. Therefore, we treated **1** with KO_2_ and 18-crown-6 to examine whether the reaction proceeds only with the superoxide. As a result, only the starting material, **1**, was recovered, which indicates that **3** is produced by a concerted reaction of the direct oxidation of **1** on the anode and the reduction of dissolved oxygen on the cathode.

**Table 2:** Control electrolysis experiments of **1**^a^.

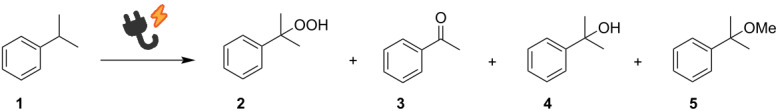						

Entry	Solvent	Isolated yields (%)				

		**1**	**2**	**3**	**4**	**5**

1	MeCN–MeOH 9:1	trace	4	trace	12	21
2	MeCN (dehydrated)	trace	1	29	15	n.a.
3	MeCN–H_2_O 9:1	n.d.	6	9	20	n.a.
4	MeCN–H_2_O 1:1	n.d.	trace	trace	15	n.a.

^a^Reaction conditions: BDD anode and cathode, 1 mmol cumene (**1**), 5 mL solvent, 0.1 M Et_4_NClO_4_, 2.1 mA/cm^2^ and *Q* of 5 F (referring to mole of **1**), undivided beaker-type cell, rt. n.d. = not detected, n.a. = not applicable.

**Figure 2:**
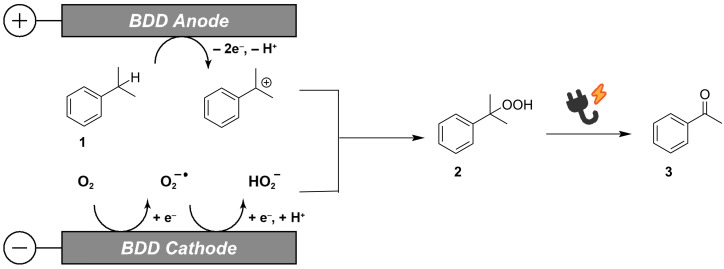
Proposed reaction mechanism of electro-conversion of cumene (**1**) into acetophenone (**3**).

Figure 2 shows a proposed mechanism. Anodic oxidation of cumene on the BDD electrode with a wide potential window preferentially affords the cumyl cation as the reaction intermediate. On the other hand, cathodic reduction of dissolved oxygen produces the superoxide and even the hydroperoxide anion. Addition of the hydroperoxide anion to the cumyl cation yields cumene hydroperoxide, which is further converted into acetophenone. This reaction pathway is supported by the following two facts. One is that cumene hydroperoxide was obtained as a byproduct, and the other is that electrolysis of cumene hydroperoxide as a starting material afforded acetophenone [18]. It should be noted that the tertiary carbon at the benzyl position is a key for the present molecular transformation, since acetophenone was yielded in 19% as the main product by the electrolysis of *sec*-butylbenzene as a starting material, while propylbenzene was not. Moreover, the electrolysis under a flow of oxygen did not improve the yields, which indicates that the BDD cathode can utilize the electrogenerated oxygen species efficiently, as we have reported previously [19].

## Conclusion

We have demonstrated a straightforward electro-conversion of cumene into acetophenone using boron-doped diamond (BDD) electrodes. The BDD’s wide potential window enabled the direct anodic oxidation of cumene to afford a key reaction intermediate, which cannot be realized by other electrodes such as graphite and Ni. Electrosynthesis is a sustainable, scalable, and cost-efficient protocol; a specific catalyst is not required, and reagent waste can be avoided. In addition, the present work offers new perspectives for an electrosynthetic strategy toward oxidation reactions of aromatic alkyls.

## Experimental

### General protocol for electro-conversion of cumene

Electrolysis was carried out by using an IKA screening system (IKA, Germany). A solution of cumene (**1**, 0.12 g, 1.00 mmol) and supporting electrolyte (0.1 M) in 5 mL solvent was transferred into the electrolysis cell equipped with electrodes (purchased from IKA, Germany; 0.3 × 1.0 × 7.0 cm; immersed 1.8 cm into solution). A constant current electrolysis was performed at room temperature. After application of the desired amount of charge, the electrolysis was stopped, and the solvent was removed in vacuo. The residue was purified by silica gel column chromatography (CH_2_Cl_2_).

## Supporting Information

File 1 Characterization data and ^1^H NMR spectra of isolated compounds **2**, **3**, **4**, and **5**.
