# Supplementary material for: Electro-conversion of cumene into acetophenone using boron-doped diamond electrodes
Source: Beilstein J Org Chem. 2022 Sep 7;18:1154–8. doi: 10.3762/bjoc.18.119 (PMC9475189; doi:10.3762/bjoc.18.119)
Supplement: File 1 — Characterization data and 1H NMR spectra of isolated compounds 2, 3, 4, and 5. [file Beilstein_J_Org_Chem-18-1154-s001.pdf]

BEILSTEIN JOURNAL OF ORGANIC CHEMISTRY

Supporting Information

for

Electro-conversion of cumene into acetophenone using boron-doped diamond electrodes

Mana Kitano, Tsuyoshi Saitoh, Shigeru Nishiyama, Yasuaki Einaga
and Takashi Yamamoto

Beilstein J. Org. Chem. **2022,** *18,* 1154–1158. doi:10.3762/bjoc.18.119

Characterization data and ^{1}H NMR spectra of isolated compounds 2, 3, 4, and 5

License and Terms: This is a supporting information file under the terms of the Creative Commons Attribution License (https://creativecommons.org/licenses/by/4.0). Please note that the reuse, redistribution and reproduction in particular requires that the author(s) and source are credited and that individual graphics may be subject to special legal provisions.
The license is subject to the *Beilstein Journal of Organic Chemistry* terms and conditions: (https://www.beilstein-journals.org/bjoc/terms)

Characterization data and ^{1}H NMR spectra of isolated compounds

<u>Cumene hydroperoxide (**2**)</u>

^{1}H NMR (400 MHz, $CDCl_3$): δ 7.49–7.24 (m, 5H), 1.62 (s, 6H).

The spectral data agreed with the literature [S1].

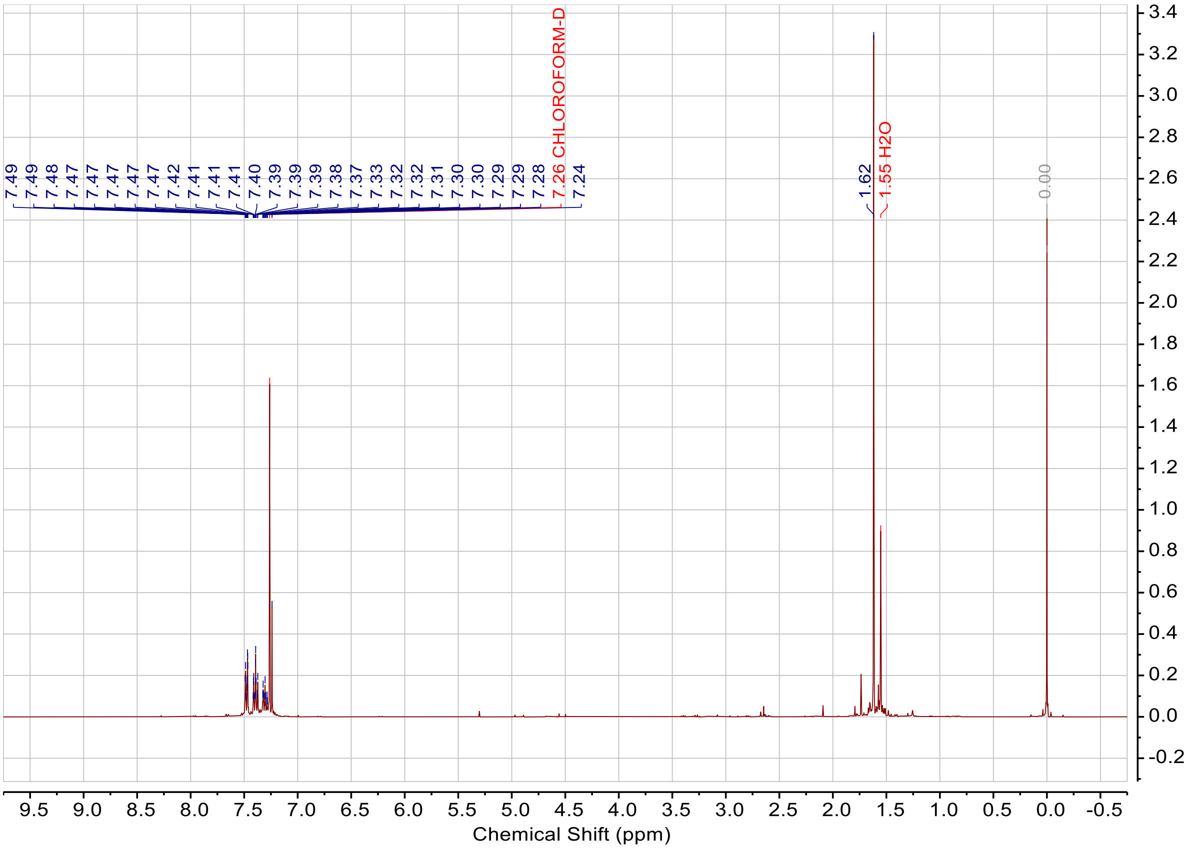

Figure S1: ^{1}H NMR spectrum of cumene hydroperoxide (**2**).

Acetophenone (**3**)

^1H NMR (400 MHz, $CDCl_3$): δ 7.97–7.44 (m, 5H), 2.61 (s, 3H).

The spectral data agreed with the literature [S1].

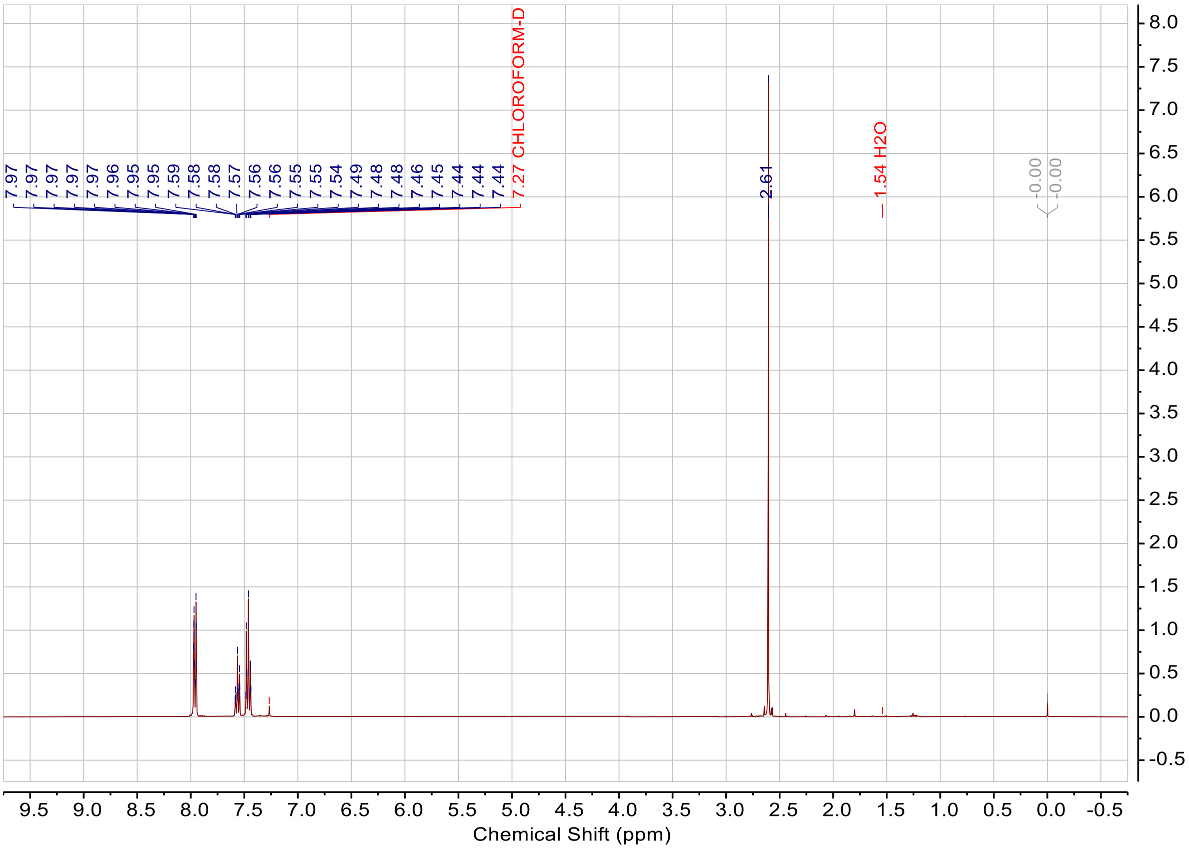

Figure S2: ^1H NMR spectrum of acetophenone (**3**).

Cumyl alcohol (**4**)

^{1}H NMR (400 MHz, $CDCl_3$): δ 7.50–7.22 (m, 5H), 1.84 (s, 1H), 1.58 (s, 6H).

The spectral data agreed with the literature [S1].

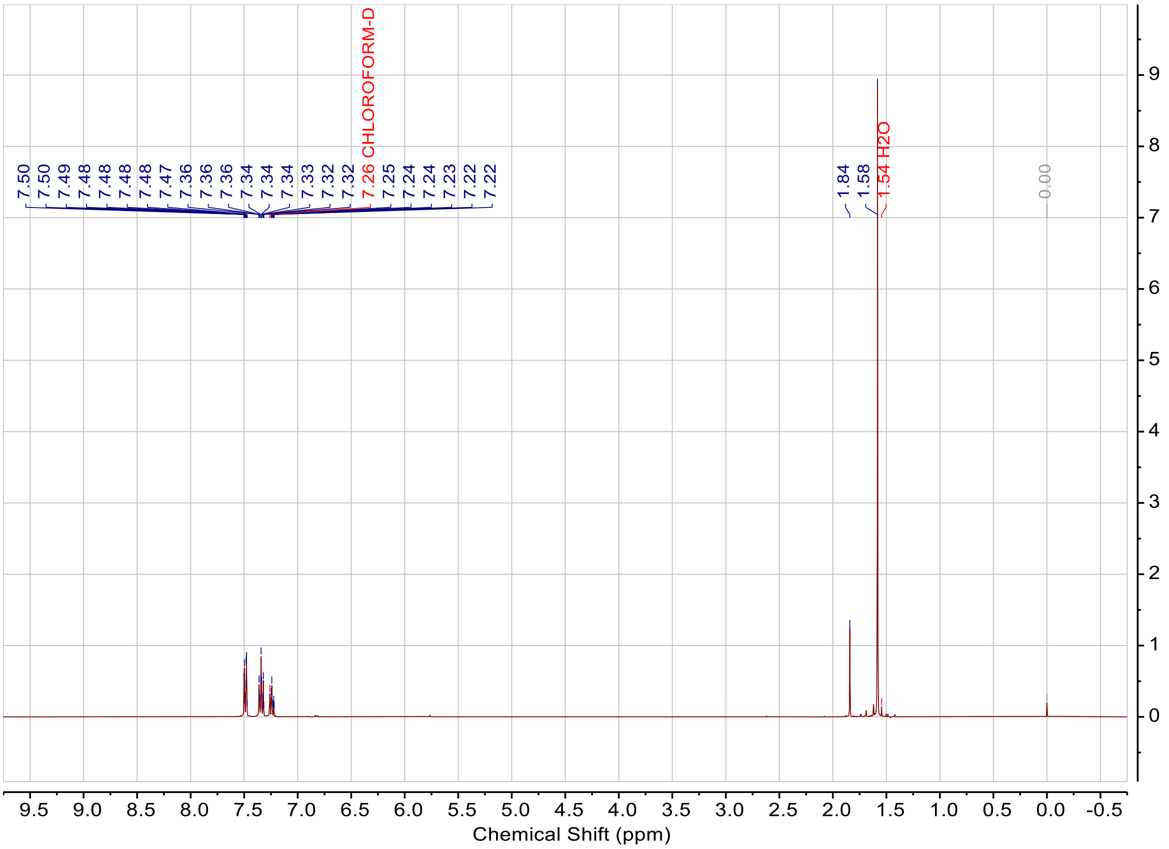

Figure S3: ^{1}H NMR spectrum of cumyl alcohol (**4**).

Methyl cumyl ether (**5**)

^{1}H NMR (400 MHz, $CDCl_3$): δ 7.48–7.22 (m, 5H), 3.07 (s, .3H), 1.53 (s, 6H).

The spectral data agreed with the literature [S2].

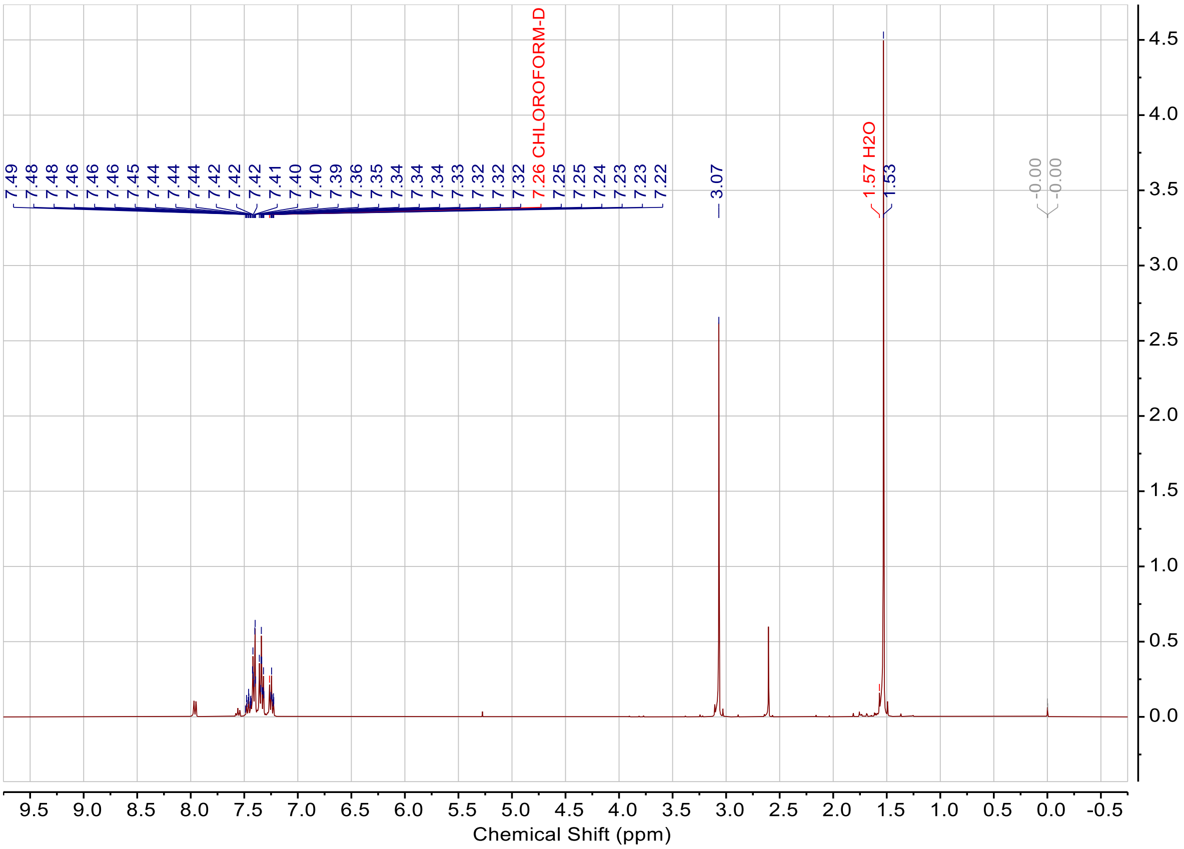

Figure S4: ^{1}H NMR spectrum of methyl cumyl ether (**5**).

References

S1. The Aldrich FT-NMR library, Sigma-Aldrich Corp., 2003.

S2. Murphy, J. A.; Khan, T. A.; Zhou, S.-Z.; Thomson, D. W.; Mahesh, M. *Angew. Chem. Int. Ed.* **2005**, *44*, 1356–1360.
